# Molecular Cloning and Characterization of Three Genes Encoding Dihydroflavonol-4-Reductase from *Ginkgo biloba* in Anthocyanin Biosynthetic Pathway

**DOI:** 10.1371/journal.pone.0072017

**Published:** 2013-08-26

**Authors:** Cheng Hua, Li Linling, Cheng Shuiyuan, Cao Fuliang, Xu Feng, Yuan Honghui, Wu Conghua

**Affiliations:** 1 Economic Forest Germplasm Improvement and Comprehensive Utilization of Resources of Hubei Key Laboratories, Hubei Huanggang, China; 2 College of Chemistry and Life Science, Huanggang Normal University, Huanggang, China; 3 College of Forest Resources and Environment, Nanjing Forestry University, Nanjing, China; Russian Academy of Sciences, Institute for Biological Instrumentation, Russian Federation

## Abstract

Dihydroflavonol-4-reductase (DFR, EC1.1.1.219) catalyzes a key step late in the biosynthesis of anthocyanins, condensed tannins (proanthocyanidins), and other flavonoids important to plant survival and human nutrition. Three *DFR* cDNA clones (designated *GbDFRs*) were isolated from the gymnosperm *Ginkgo biloba*. The deduced GbDFR proteins showed high identities to other plant DFRs, which form three distinct DFR families. Southern blot analysis showed that the three *GbDFRs* each belong to a different DFR family. Phylogenetic tree analysis revealed that the *GbDFRs* share the same ancestor as other DFRs. The expression of the three recombinant GbDFRs in *Escherichia coli* showed that their actual protein sizes were in agreement with predictions from the cDNA sequences. The recombinant proteins were purified and their activity was analyzed; both GbDFR1 and GbDFR3 could catalyze dihydroquercetin conversion to leucocyanidin, while GbDFR2 catalyzed dihydrokaempferol conversion to leucopelargonidin. qRT-PCR showed that the *GbDFRs* were expressed in a tissue-specific manner, and transcript accumulation for the three genes was highest in young leaves and stamens. These transcription patterns were in good agreement with the pattern of anthocyanin accumulation in *G.biloba*. The expression profiles suggested that *GbDFR1* and *GbDFR2* are mainly involved in responses to plant hormones, environmental stress and damage. During the annual growth cycle, the *GbDFRs* were significantly correlated with anthocyanin accumulation in leaves. A fitted linear curve showed the best model for relating *GbDFR2* and *GbDFR3* with anthocyanin accumulation in leaves. *GbDFR1* appears to be involved in environmental stress response, while *GbDFR3* likely has primary functions in the synthesis of anthocyanins. These data revealed unexpected properties and differences in three DFR proteins from a single species.

## Introduction

Flavonoids are a large family of plant secondary metabolites, that exist widely throughout the plant kingdom. Flavonoids are divided into several structural classes, including flavanones, isoflavonoids, flavonols, anthocyanins, catechins, and condensed tannins, and are abundant in fruits, leaves and flowers [1]. To date, over 9000 different flavonoid compounds have been identified [2], many of which are involved in biological processes such as pigmentation of flowers, auxin transport regulation, seed development, protection against UV-B damage, defense against pathogens and pests, and pollen viability [3]–[5]. Several flavonoids are active ingredients in herbal medicines and appear to confer health benefits to humans when consumed regularly [6]. Particular attention has been placed on the anthocyanins, catechins, and proanthocyanidins because of their antioxidant activities and their interactions with human health [6], [7]. The biosynthetic pathway of flavonoids are well established in plants. Many flavonoid biosynthetic genes are induced under stress conditions and, accordingly, flavonoid levels increase during exposure to biotic and abiotic stresses, such as wounding, drought, metal toxicity and nutrient deprivation [8], [9].

Dihydroflavonol 4-reductase (**DFR**, EC1.1.1.219) is the first committed enzyme of the flavonoid biosynthetic pathway leading to common anthocyanins. DFR is a rate-limiting enzyme in the biosynthesis of anthocyanins and condensed tannins and catalyzes the reduction of dihydroflavonols to leucoanthocyanins [10]. DFR preparations from several plants can catalyze the reduction of three dihydroflavonols, kaempferol (DHK), dihydroquercetin (DHQ) and dihydromyricetin (DHM), into leucoanthocyanidins, which are common precursors for anthocyanin and condensed tannin synthesis (Figure S1) [11], [12]. The genetics and regulation of DFR have been studied extensively in various plants [10], [11], [13], [14]. Induction of DFR activity has been linked to an increase in condensed tannins accumulation, which may be important for defense against herbivores [15]. Knowledge of the details of DFR biochemistry is very important for understanding aspects of flavonoid biosynthesis, especially how plants regulate flavonoids, condensed tannins synthesis and anthocyanin biosynthesis. Little has been reported regarding other biochemical properties of DFR isoenzymes, and the role of isoforms in the regulation of specific branches of the flavonoid pathways is unclear.

Today, *Ginkgo biloba* is one of the most popular functional plants, especially as a medicinal plant. Extracts of *G.biloba* leaves contain active compounds, such as flavonoids and terpene lactones (ginkgolides and bilobalide), and can be used to increase peripheral and cerebral blood flow [16],[17]. The flavonoid and tannin metabolisms are both branches of phenylalanine metabolism, and tannin synthesis might affect the accumulation of *G. biloba* flavonoids. Early *in vitro* enzyme assays using cell-free extracts of *G. biloba* and *Pseudotsuga menziesii* showed that DFR converts DHQ to leucocyanidin and DHM to leucodelphinidin (Figure S1) [11], [18]. Limited information has been provided by recent recombinant expression studies of DFR *in vitro*. And little has been reported regarding other biochemical properties of DFR isoenzymes, and their roles in the regulation of specific branches of the flavonoids pathway [11]. In this study, we isolated three full-length cDNAs(*GbDFRs*), *GbDFR1*, *GbDFR2* and *GbDFR3*, encoding DFR isoenzymes from the *G. biloba* EST database. The expression profiles of the *GbDFRs* were investigated in various tissues of *Ginkgo*, and their potential biological functions in the flavonoid and anthocyanin biosynthetic pathways were investigated.

## Materials and Methods

### Plant Materials and Treatments

Fourteen-year old of grafted *G. biloba* seedlings were growing in a greenhouse in Huanggang (E, 111°54′–112°19′, N, 30°6′–30°39′, Hubei province, central of China) were sampled as plant materials. For gene cloning and tissue expression, diverse tissues including young leaves, mature leaves, ovules, stamens, albumen, gynoecia, stems and roots were collected for DNA and RNA extraction as described by Xu et al. [19]. Tissues were immediately frozen in liquid nitrogen and kept at −80°C prior to total RNA extraction.

One year old cuttings from the same genotypic strain of *G. biloba* were subjected to treatments with UV-B, wounding (WOU), 5-aminolevulinic acid (ALA), abscisic acid (ABA), salicylic acid (SA) and ethephon (ETH). For UV-B treatment, seedlings were exposed to 1500 µJ/m^2^ UV-B irradiation in a closed chamber, and the control cuttings were placed in a dark closed chamber. The edges of *Ginkgo* leaves were cut by about 0.6 cm with scissors for wounding treatment, the intact leaves of *Ginkgo* was as control. The ALA (100 µM), ABA (10 mM), SA (25 mM), and ETH (20 mM) were dissolved in 0.01% Tween 20 and sprayed onto young leaves. The control leaves were sprayed with an equivalent volume of 0.01% (v/v) Tween 20.

### Southern Blot Analysis

Genomic DNA (20 µg/sample) was digested overnight at 37°C with *Sma* I, *EcoR* V, *Sac* I, *Kpn* I, *Pvu* II, *Xma* I, *Dra* I, *Cla* I and *Hind* III. The digested DNA was fractionated by 0.9% agarose gel electrophoresis and transferred onto a PVDF membrane (Roche Applied Science, Germany). At total of 50 ng purified GbDFRs was used as a template in a total volume of 20 µL for probe labeling. Probe labeling (DIG), hybridization and signal detection were performed following the manufacturer’s instruction for the DIG High Primer DNA Labeling and Detection Starter Kit II (Roche Applied Science, Germany).

### Isolation of *G. biloba* cDNA Clones Encoding GbDFR1, GbDFR2 and GbDFR3 Protein

One EST sequence with high BLASTX scores against sequences of known DFR clones was designated GbDFR1. A second EST with high BLASTX scores against known DFR sequences was designated GbDFR2. The third EST with high BLASTX scores against known DFR sequences was designated GbDFR3. Base on the EST sequence, the specific primer pair (1F5R1, 2F5R1, 3F5R1 and 1F3R1, 2F3R1, 3F3R1) and the nested primer pair (1F5R2, 2F5R2, 3F5R2 and 1F3R2, 2F3R2, 3F3R2) were designed to amplify the 5′ and 3′ end of *GbDFRs* using the SMART™ RACE cDNA Amplification Kit (Clontech, Mountain View, CA, USA). Table 1 lists the primer sequence for each gene. The PCR products were purified and cloned into the pMD18-T vector for sequencing. After comparing and aligning the sequence of 5′ RACE, 3′ RACE, and the internal fragment, the full-length cDNA sequence of *GbDFRs* were obtained. The full-length cDNA of *GbDFR1*, *GbDFR2* and *GbDFR3* were obtained when the 5′ and 3′ fragments were assembled by Vector NTI 10.0 software.

**Table 1:** The gene clone and analysis primers of *GbDFRs*.

Primer	Sequence (5′ to 3′)	Description
1F5R1	CCGATGATCTTGGTTTTCCTGCAGT 247	GbDFR1 Reverse primer for 5′ RACE, outer
1F5R2	ACATAACTGCTGAAACGGAAG	GbDFR1 Reverse primer for 5′ RACE, nested
1F3R1	AGTTTTCCACGTCGCTAGTCCTGTT	GbDFR1 Forward primer for 3′ RACE, outer
1F3R2	GAGAGGTTGTGGGTCCTGCTG	GbDFR1 Forward primer for 3′ RACE, nested
1FZ1	*ACAGGATCC * ATGGCATGCTCAAGTTCGAAG	Prokaryotic expression, forward
1FZ2	*CAACTCGAG * TTACAGAAGGCCTCTTGCTTTGT	Prokaryotic expression, reverse
1FT1	AATACATACTTCTTCCGTTTCAG 423	GbDFR1 Primer for qRT-PCR, forward
1FT2	GCTATGAAATACATCCAGCCGAT 557	GbDFR1 Primer for qRT-PCR, reverse
GAPU	TAGGAATCCCGAGGAAATACC	GAPDH Primer for qRT-PCR, forward
GAPD	TTCACGCCAACAACGAACATG	GAPDH Primer for qRT-PCR, reverse
2F5R1	TTGGGCCAATTACAAATGTAGGAAG	GbDFR2 Reverse primer for 5′ RACE, outer
2F5R2	CTGCTTTCTCTGCTAATGTCTTG	GbDFR2 Reverse primer for 5′ RACE, nested
2F3R1	GTGTGACTGGAGCCTCTGGTTACTT	GbDFR2 Forward primer for 3′ RACE, outer
2F3R2	TACAACAAGGTTACTATGTCAGGG	GbDFR2 Forward primer for 3′ RACE, nested
2FZ1	*ACAGGATCC * ATGGAGGCCGAACCTGAAAAC	Prokaryotic expression, forward
2FZ2	*CAACTCGAG * TCAAGGAGCAAGGCCACCTTTAA	Prokaryotic expression, reverse
2FT1	TCAATGGGTTTAGAGGAACGGTA	GbDFR2 Primer for qRT-PCR, forward
2FT2	GTTCCCCTGACATAGTAACCTTG	GbDFR2 Primer for qRT-PCR, reverse
3F5R1	GAACAAGACTTGAGCACATTGAGTG	GbDFR3 Reverse primer for 5′ RACE, outer
3F5R2	ACAGCAGGGTCAATTAGGGTTTC	GbDFR3 Reverse primer for 5′ RACE, nested
3F3R1	AGTTTTCCATACTGCATCTCCTGTC	GbDFR3 Forward primer for 3′ RACE, outer
3F3R2	CATAGTACCATACAATGAGCACCT	GbDFR3 Forward primer for 3′ RACE, nested
3FZ1	*ACAGGATCC * ATGAGCGAGGTGTGTGTGACAG	Prokaryotic expression, forward
3FZ2	*CAACTCGAG * TCAATTATGTTTATGATGATCAGGG	Prokaryotic expression, reverse
3FT1	TCATAGCCGCTTATCTTATTCGC	GbDFR3 Primer for qRT-PCR, forward
3FT2	TACAATCTTCAATCTCTCGGTGG	GbDFR3 Primer for qRT-PCR, reverse

The underlined nucleotides are restriction enzymes cleavage sites.

### Expression of GbDFRs Proteins and *in vitro* Enzyme Assay

To clone *GbDFRs* into an expression vector, three pair of primers (Table 1, 1FZ1, 1FZ2, 2FZ1, 2FZ2, 3FZ1, 3FZ2) were designed and synthesized to amplify the coding region by RT-PCR with the incorporation of a restriction enzyme site and a protective base to simplify later vector construction. After confirmation by sequencing, the resulting recombinant plasmid was introduced into BL21 (DE3) by the heat shock method. A single colony of *E.coli* BL21 cells harboring the expression plasmid pET28a-GbDFRs was inoculated at 37°C in Luria-Bertani medium containing kanamycin (50 mg·L^−1^) and were grown with shaking (150 rpm) at 37°C until the optical density (OD 600) reached about 0.6. For induction, Isopropyl β-D-thiogalactoside (IPTG) was added at a final concentration of 1 mM and the cells were further cultured at 30°C for 2 h. The cells were lysed by sonication for 10 s at 4°C, and centrifuged at 7000 g for 15 min. Supernatants and pellets were analyzed by SDS-PAGE and Coomassie Brilliant Blue R250 staining. The recombinant GbDFRs protein from induced cells was purified using Nickel-CL agarose affinity chromatography (Bangalore Genei) and used for *in vitro* enzyme assays.

Western blotting was carried out to verify expression of GbDFRs protein having a His-tag in the N-terminus. After electrophoresis, the proteins were electro-transferred onto a PVDF membrane and detected with antimouse RGS-His antibody (Santa Cruz, American). The purified antibody, which was dissolved to 10 µg·mL^−1^ in 50 mM carbonate salt vuffer (pH 9.6), was coated on immunoplates at 100 µL aliquot per well at 37°C for 2 h then at 4°C for 48 h. the goat antibody against a mouse IgG, conjugated with alkaline phosphatase (AP), was used as the second antibody. Western Blue Stabilized Color Substrate for AP (Promega, USA) was used for the color reaction.

### High Performance Liquid Chromatography Analysis of Enzyme Reaction Products

Dihydroflavonols including (±)-taxifolin (DHQ; Sigma) and Dihydrokaempferol (DHK; Apin, Abingdon, Oxon, UK) were dissolved in methanol at 10 mg·mL^−1^. Reactions for GbDFR1 were conducted at 30°C for 30 min using 370 µL of 100 mM Tris-HCl buffer (pH7.0), 70 µL of GbDFR1 or GbDFR3 enzyme extract, 10 µL of substrate, and 50 µL of 20 mM NADPH, whereas reactions for GbDFR2 substituted 370 µL of 50 mM citrate/phosphate buffer (pH 6.2), so that enzymes were assayed at their respective optimum pH values.

Compounds were resolved on a C18-silica High Performance Liquid Chromatography (HPLC) column (5 µm, narrow pore, 4.6×250 mm, Bakerbond, J.T. Baker, Phillipsburg, NJ, USA) with detection at 280 nm, the maximum absorbance wavelength for most of the substrates and products. UV spectra were recorded with a UV diode array detector (model 168, Beckman Instruments). For routine HPLC quantitation, the solvents were 1% (v/v) H_3_PO_4_ in water (A) and methanol (B), and separation methods were developed by adapting the systems reported previously [20]. For initial DFR assays or screening of possible substrates, the column was equilibrated at 15% (v/v) B at a flow rate of 1 mL·min^−1^. Following injection (20 µL), a linear gradient from 15% to 60% (v/v) B in 20 min was initiated. Peaks were identified by retention time and UV spectra, compared with standards available in house. For later assays using only taxifolin, the assay time was reduced by increasing the flow rate to 1.5 mL min^−1^ and decreasing the gradient to 8 min.

### Quantitative Real-Time PCR Analysis of Transcript Levels

The transcription levels of *GbDFRs* were determined in different *G. biloba* tissues, as well as in young seedling leaf samples collected at different time points after stress or hormone treatments. Quantitative Real-Time PCR (qRT-PCR) was carried out using an ABI PRISM 7500 Sequence Detection System (Applied Biosystems, American) with SYBR Green PCR Master Mix (Applied Biosystems, American) according to the manufacturer’s protocol. The *G. biloba* glyceraldehydes-3-phosphate dehydrogenase gene (*GbGAPDH*, L26924) [20], was used as the reference gene as described by Xu [19].

The three pair of gene-specific primers (1FT1, 2FT1, 3FT1 and 1FT2, 2FT2, 3FT2) and reference primers (GAPU, GAPD) for QRT-PCR were listed in Table 1. The qRT-PCR conditions were: 10 min at 95°C, and 40 cycles (95°C for 15 s, 60°C for 1 min). Before performing qRT-PCR, primer efficiency was evaluated using both *GbDFRs* and *GbGAPDH* at 100, 150, 200, 250 and 300 nM combinations. A 120 nM concentration was chosen as most suitable combination for both genes. The efficiency of these primers was investigated by applying primer melting curve analysis and gel electrophoresis. Both results indicated that each primer pair gave a specific and unique product. For each plant sample, aliquots of 150 ng total RNA was analyzed for each gene and the four genes (*GbDFRs* and *GbGAPDH*) were always analyzed simultaneously. Each sample was amplified 3 times and all reactions were performed on ABI PRISM 7500 Sequence Detection System. With a housekeeping gene *GbGAPDH*, the relative amount of the *GbDFRs* transcript is presented as 2^ (−ddCt)^ according to the C_T_ method (dCt = Ct_sample_-Ct_control_) described in the qRT-PCR Application Guide (Applied Biosystems). When comparing the expression of *GbDFRs* in different tissues or treatments, the relative expression of *GbDFRs* was achieved by calibrating its transcription level to that of the reference gene, *GbGAPDH*.

### Analysis of Anthocyanins in *G. biloba*

Anthocyanin quantification was performed as described by Pang et al [21]. The *Ginkgo* tissues were ground in liquid nitrogen and placed into 5 mL extraction buffer (an equal volume of methanol: 0.1% HCl), sonicated for 1 h and then shaken in darkness for 4 h at 120 rpm. After centrifugation at 2600 g for 12 min, 1 mL of water was added to 1 mL of extract, followed by the addition of 1 mL of chloroform to remove chlorophyll. The absorption of the extracts was measured spectrophotometrically at 530 nm. The amount of anthocyanins was reported as 10× (A_530_)·g^−1^ fresh weight (FW). The experiment was repeated three times for each treatment.

### Bioinformatics Analysis and Molecular Evolution Analyses

The obtained sequences were analyzed using bioinformatics tools at websites (http://www.ncbi.nlm.nih.gov and http://www.xpasy.org). Vector NTI Suite 10 was used for DNA sequence alignment and analysis. Amino acid sequence alignments were performed with DNAMAN software (Lynnon Corporation, USA). Phylogenetic tree analysis of *GbDFRs* and known *DFRs* from other plant species retrieved from GenBank were aligned with Mega 4.0 [22]. The phylogenetic tree was constructed by a neighbor-joining (NJ) method and measured by bootstrap analysis with 1000 replicates. Statistical differences in expression between the mean values of the control and tested samples were analyzed by one-way analysis of variance (ANOVA) and the multiple linear regression, SPSS 17 was used for statistical analysis and graphing.

## Results

### Characterization of *G. biloba* DFR cDNA Clones

**Figure 1:**
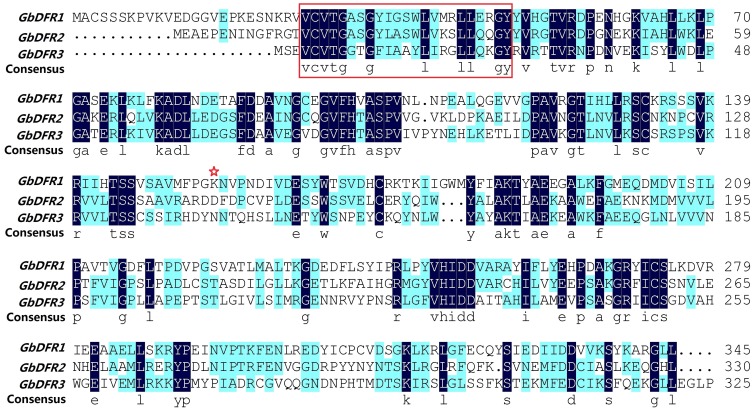
Alignment of the amino acid sequences encoded by GbDFR1, GbDFR2 and GbDFR3. Residues that are identical in the three sequences are marked with an dark background. The boxed region represents a putative NADPH binding domain at the N terminus of GbDFR proteins. The red star over residue 134 in GbDFRs indicates the Asn, Asp or no Asn/Asp residue that has a major impact on the utilization of DHK or DHQ.

By searching the blastx results from expressed sequence tags generated from a *G. biloba* cDNA library, we identified three different cDNA clones with high similarity to other DFRs, designated *GbDFR1*, *GbDFR2* and *GbDFR3*. The *GbDFR1* cDNA contained 1303 nucleotides, including a full-length open reading frame encoding 345 amino acids. The second DFR cDNA clone contained a truncated ORF, we obtained a clone containing the full-length GbDFR2 ORF (encoding 340 amino acids) and the 3′5′-untranslated region totaling 1276 necleotides. The GbDFR3 cDNA contained 1420 necleotides, including a full-length ORF encoding 333 amino acids (Figure S2–S4, Figure 1).

To examine if the *GbDFRs* belongs to a multigene family, aliquots of 20 µg genomic DNA were digested with *Sma* I, *EcoR* V, *Sac* I for *GbDFR1*, *Kpn* I, *Pvu* II, *Xma* I for *GbDFR2* and *DraI*, *Cla* I and *Hind* III for *GbDFR3*. The restriction site did not cut within the coding region. These fragments were then hybridized with the coding sequence of *GbDFRs* under high stringency conditions. In total, 4–5 specific hybridization bands ranging from 0.5 kb to 10 kb were recovered in each of the restriction enzyme-digested lanes (Figure S5), this indicated that *GbDFRs* belonged to a multigene family.

**Figure 2:**
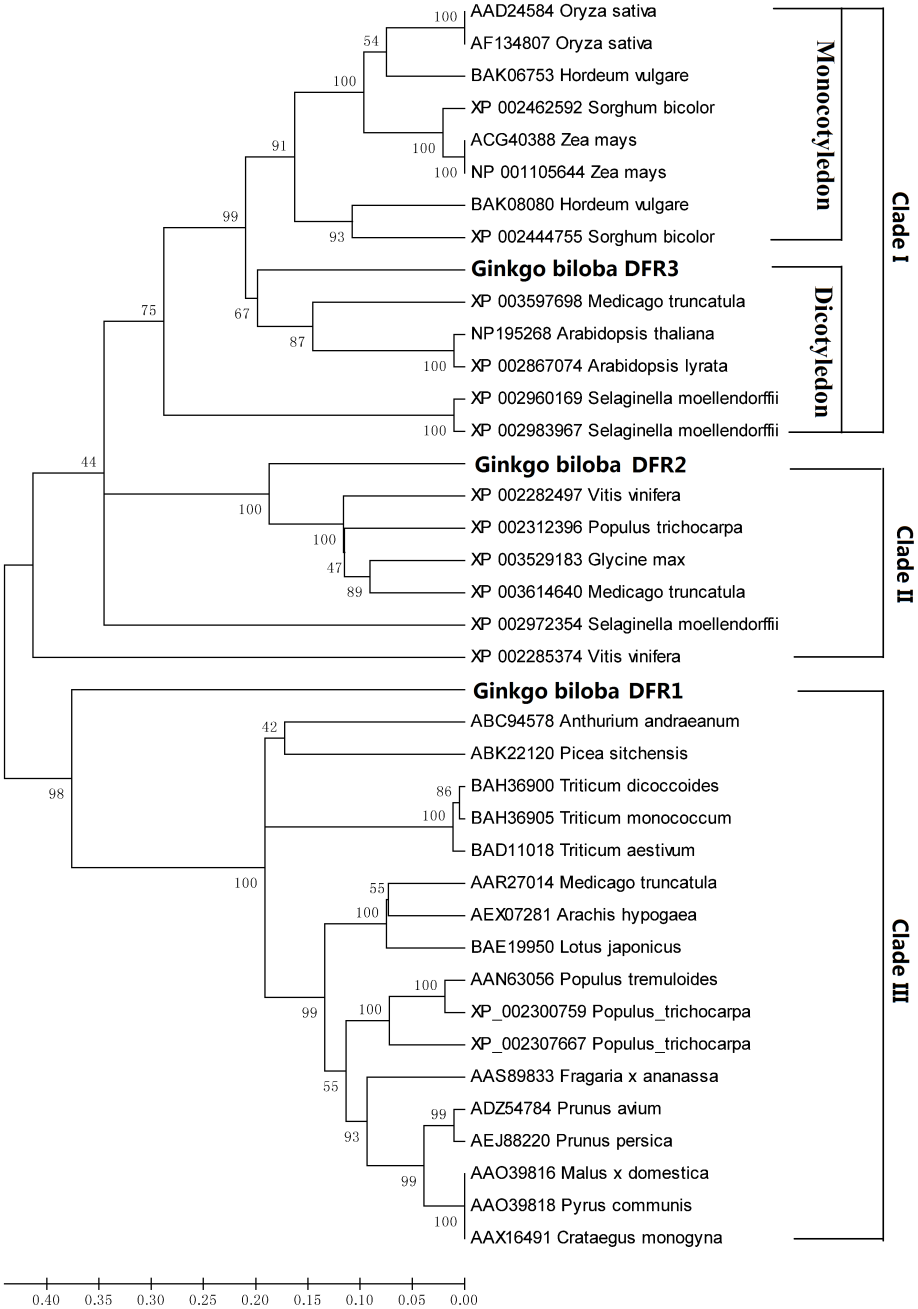
Phylogenetic tree of plant chloroplast *GbDFR*s. The tree was constructed by the Neighbor-Joining method, MEGA4.1. The numbers at each node represented the bootstrap value, with 1000 replicates.

To further investigate the sequence homology of GbDFRs amino acid sequence to other known DFRs, a phylogenetic tree generated by the neighbor-joining method based on the putative amino acid sequences of DFRs showed that all members of the DFR family could be sorted into three different monophyletic clades. (Figure 2).

### Functional Expression in *E.coli* and *in vitro* Biochemical Characterization

Although several *DFR* genes have been identified by genetic studies, and DFR cDNA have been characterized and expressed in plants, little has been published regarding the biochemical characterization of the proteins encoded by these clones, especially multiple DFR enzymes from the same species. To functionally characterize the three *G.biloba* DFR enzyme, we subcloned the coding regions of *GbDFR1*, *GbDFR2* and *GbDFR3* into pET28a and expressed the recombinant proteins in *E.coli* strain BL21 (Figure S6, S7 and S8). Soluble protein extracts from IPTG-induced cels were prepared and assayed for enzyme activity using methods previously used for GbCHI and GbFLS [8], [23].

**Figure 3:**
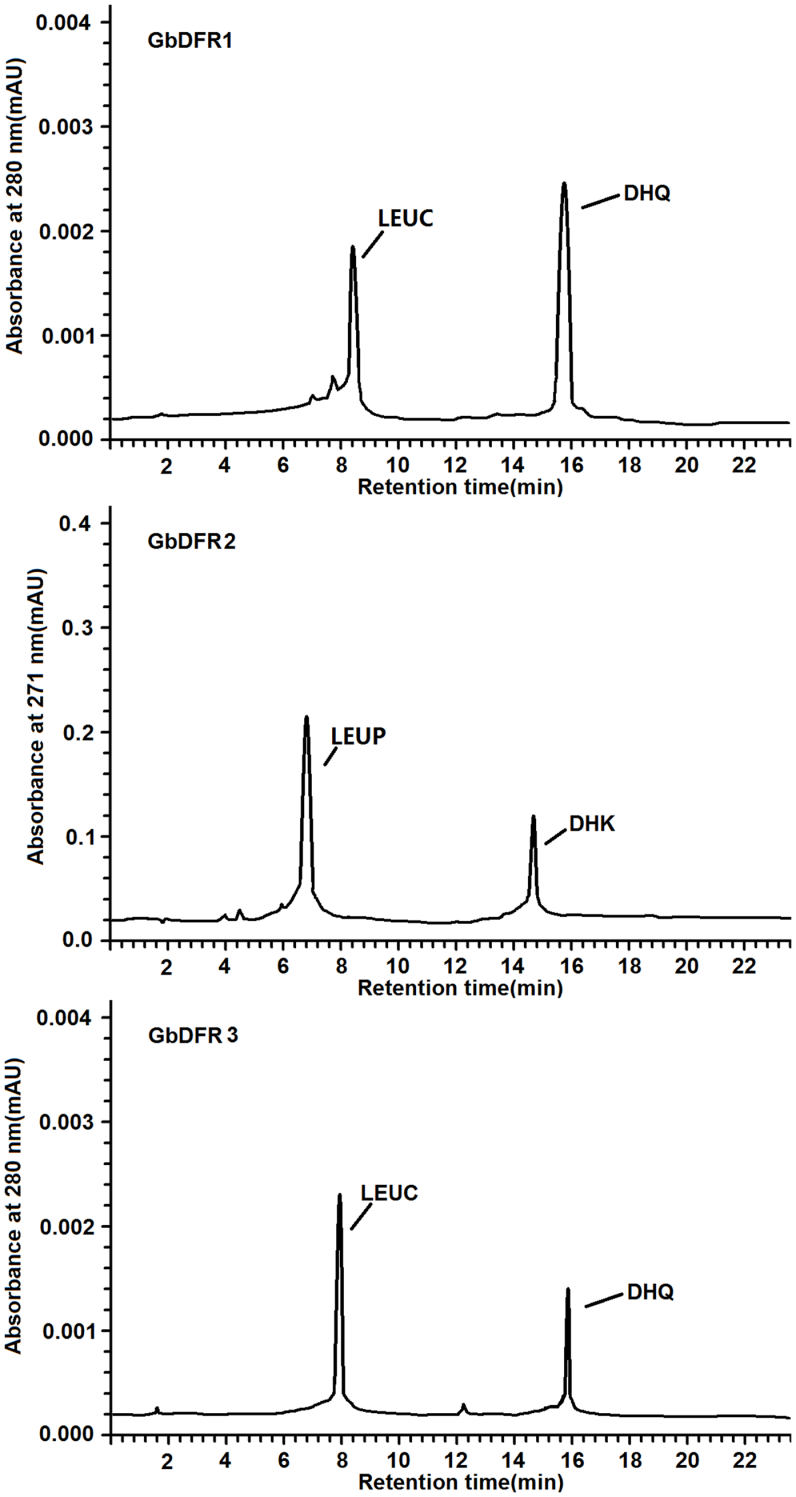
HPLC chromatograms of GbDFRs enzyme assay extracts. Assay mixtures contained DHQ and DHK as substrate, NADH, and protein extracts from *E. coli* harboring BL21-GbDFR1, BL21-GbDFR2 and BL21-GbDFR3. Chromatograms were recorded at the UV absorbance wavelength of 280 nm and 271 nm. The identity of the leucocyanidin product was confirmed based on relative retention time, UV spectra.

The most common dihydroflavonol in plants is DHQ (or taxifolin) with two hydroxyls in the B ring. Thus, we chose taxifolin and DHK to develop an *in vitro* enzyme assay. Because of reports of instability of DFRs *in vitro* during purification, we used crude bacterial protein extracts as the enzyme source to minimize enzyme manipulations. Enzyme extracted from cultures expressing either GbDFR1 and GbDFR3 protein converted DHQ to a product eluting earlier in the HPLC system, whereas this product did not accumulate in reactions carried out with protein extracted from cultures harboring the GbDFR2 protein and the empty pET28a expression vector (Figure 3). When DHK was used as the substrate, enzyme extracted from cultures expressing of GbDFR2 protein converted substrate to a new peak of leucucopelargonidin, whereas this product did not accumulate in reactions carried out with GbDFR1, GbDFR3 or the empty pET28a expression vector (Figure 3).

### The Expression of the *GbDFRs* and Accumulation of Anthocyanin in Different Tissues

**Figure 4:**
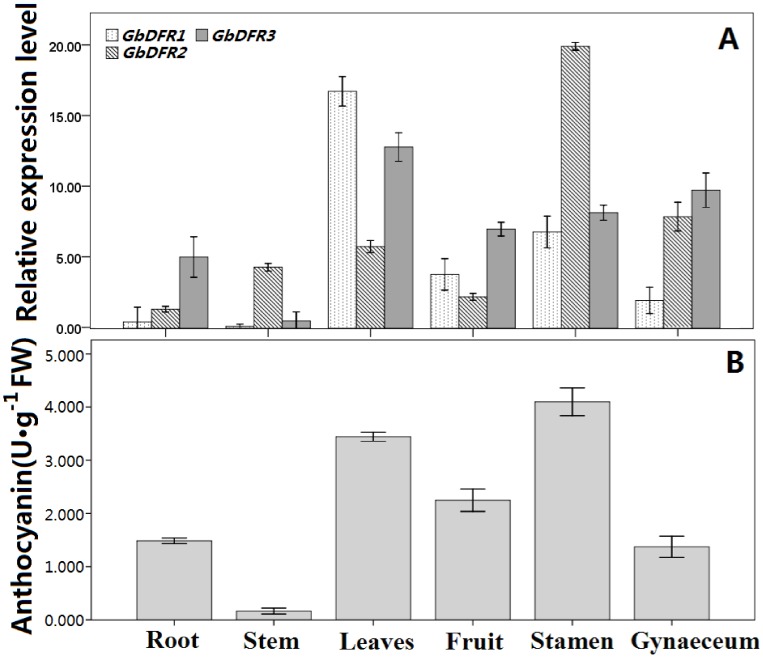
Comparison among different tissues in *G. biloba* regarding relative amount of *GbDFRs* mRNA and accumulation of anthocyanin. A: Expression pattern of *GbDFRs* gene in different tissues with *GAPDH* gene as control. B: Accumulation pattern of anthocyanin in different tissues. Data are mean values of triplicate tests ± S.D.

The tissue specificity of the expression of the three DFR genes was initially examined by qRT-PCR analysis. In this study, the expression levels of the GbDFRs genes in various tissues were analyzed by quantitative real-time PCR by using genes specific primers that can distinguish the three highly similar GbDFRs transcripts. A *Ginkgo* GAPDH gene, amplified with the primers GAPU and GAPD giving a product of 171 bp, was used as a reference for loading normalization. RNA was extracted from a variety of *G. biloba* tissues, especially those accumulating anthocyanins, known products fo DFR activity. The qRT-PCR analysis results indicated that *GbDFR2* transcripts were highly abundant in stamens tissues. In contrast, *GbDFR1* and *GbDFR3* were relatively lower in stamen tissues, whereas both the two genes showed high transcripts levels in leaves, stamen and gynaeceum. Low transcript levels were observed in root and stem (Figure 4A). These results agreed with the pattern of anthocyanin accumulation in *G. biloba*. As seen in Figure 4B, anthocyanin was detected abundantly in mature stamens, and to a lesser extent in the leaves and fruits, few detected in stem. Thus, transcription level of the *GbDFRs* appears to be one of the critical factors detemining the anthocyanin accumulation pattern in *G. biloba*.

### Effect of UV-B, SA, Wouding, ABA, ETH, and ALA on *GbDFRs* Expression

**Figure 5:**
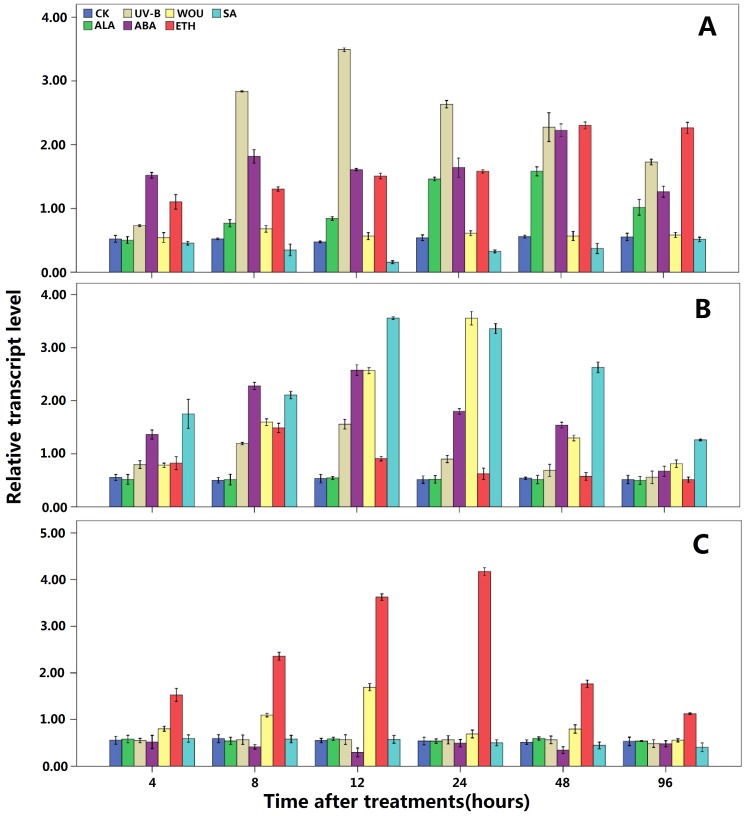
Relative quantities of *GbDFRs* mRNA by various treatment. The time points post-treatment were Wounding(WOU), ultraviolet (UV-B), Abscisic Acid (ABA), Salicylic Acid (SA), Ethephon(ETH), 5-Aminolevulinic Acid (ALA). Each sample was individually assayed in triplicate. Values shown represent the mean reading from three treated plants and the error bars indicated the mean values of triplicate tests±SD. A: The relative expression levels of *GbDFR1* at different times after the treatment. B: *GbDFR2* expression levels. C: The relative expression levels of *GbDFR3* at different times after the treatment.

Previous studies have shown that wounding was effective to the accumulation of leaves flavonoids in *Ginkgo*, whereas, the wounding treatment did not appear to induce the transcription of *GbDFR1* (Figure 5A). In response to the wounding, *GbDFR2* showed a higher stress response (Figure 5B). At 24 h after the treatment, the transcription level reached maximum, about 6.2 times the control level; and then it had dropped to 1.5 times the control level. The transcription level of *GbDFR3* rose significantly at 8 h after the treatment, reached its maximum, about 2.9 times the control at 12 h after the treatment (Figure 5C, Table S1).

When under UV-B treatment, the transcription of *GbDFR1* of the samples under the treatment of low intensity UV-B increased significantly, The transcription level of *GbDFR1* continued to rise, and reached the highest transcription level at 12 h, that’s about 7.1 times the control level. Since then the transcription began to decline slowly for a longer time (Figure 5A). The UV Treatment had the induction effect on *GbDFR2* as same. But the rise of the transcription was lower (Figure 5B). The transcription reached the maximum at the 12 h sampling point, and it’s about 2.9 times the control. After that the transcription began to decline and eventually to the control level. Compared to the above-mentioned *GbDFRs* gene, the low-dose UV treatment had no obvious induction effect on *GbDFR3* (Figure 5C).

Changes in endogenous hormones and flavonoid content are relevant in the flavonoids anabolism in *G. biloba* [24]. The treatment of ABA could significantly induce the rose of the transcription level of *GbDFR1*, but with some fluctuations. The transcription level of *GbDFR1* reached the maximum of all sampling points at the 48 h sampling point, it’s about 4.8 times the control level. After that the level began to drop (Figure 5A); ABA could raise the expression of *GbDFR2* also, the level reached the maximum at the 12 h sampling point, it’s about 5.2 times the control level. After that the transcription level began to drop slowly (Figure 5B). ABA had a down-regulated effect on the expression level of *GbDFR3* (Figure 5C, Table S1).

ALA does not belong to plant hormone, but there were reports indicated that appropriate spray of ALA can make the apple color deepened [25]. The treatment of ALA on *G. biloba* only had significant induction effect on *GbDFR1*, and no significant induction effect on *GbDFR2* and *GbDFR3*(Figure 5, Table S1).

Previous study showed that ETH treatment can improve the accumulation of *Ginkgo* flavonoids. The fundamental mechanism is that ETH improved the expression level of the key gene of flavonoid metabolism in *Ginkgo* [8], [24], [26]–[30]. It could also improve the transcription levels of these three kinds of *DFR* genes that treated the *Ginkgo* seedlings with ETH. But there’s difference in the modes of transcriptional regulation (Figure 5, Table S1).

SA regulate the development of the plant, and it’s also engaged in the pathogen resistance as a kind of endogenous signal in plant [31]. Previous research showed that the SA treatment can significantly improve the expression of *Ginkgo* flavonoids related genes [26], [27]. As flavonoid and anthocyanidin synthesis related genes, the transcription level of *GbDRF1* was inhibited by exogenous SA (Figure 5A). Whereas, *GbDFR2* was induced strongly by SA (Figure 5B). Unlike the former two, SA treatment had no significant effect on the transcription level of *GbDRF3* (Figure 5C, Table S1).

### Accumulation of Anthocyanins and the Transcription of *GbDFRs* in *Ginkgo biloba*

**Figure 6:**
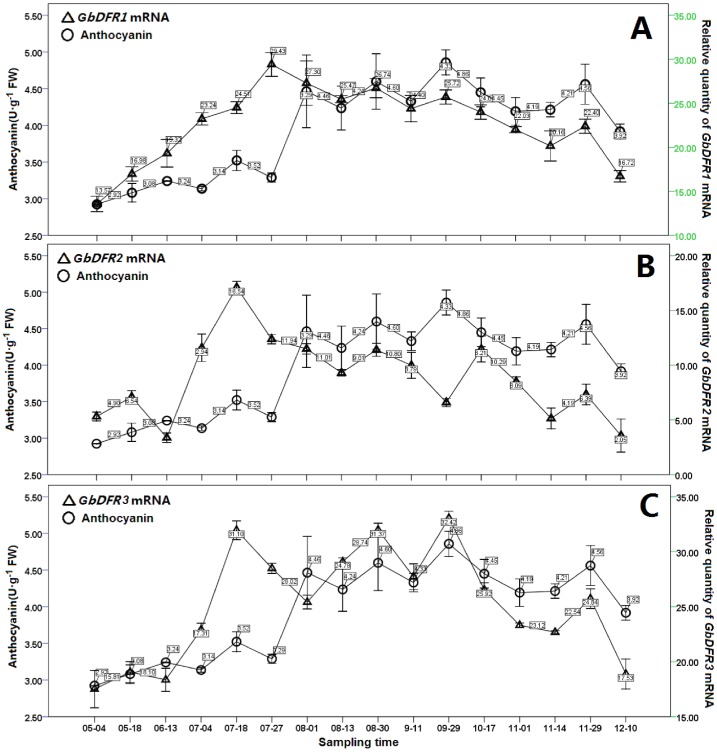
Changes in total anthocyanin content, *GbDFR1* expression levels during growth of *Ginkgo* leaves. A: The relative expression levels of GbDFR1 in different sampling time. B: The relative expression levels of GbDFR2 in different sampling time. C: GbDFR3 expression levels in different sampling time. Values shown represent the mean reading from sampling test and the error bars indicated the mean values of triplicate tests±SD.

It can be seen from Figure 6, there were several peak values in the annual variation of anthocyanin content. Obvious peak values appeared at August 1, August 30, September 29 and November 29, and the relative content was 4.46, 4.60, 4.86 and 4.56 U·g^−1^ respectively. The peak of the year appeared at the September 29 sampling point. The content change of total anthocyanin in *Ginkgo* showed a trend of first rise in the stable and then drop.

The expression of *GbDFR1* gene showed 4 peak values in the growth process of *Ginkgo*. The first appeared at the July 27 sampling point, and it was also the peak of the year, and the relative transcript level was 29.43; and then it dropped to the first low valley which appeared at Aug 13. And then it rose slightly, and showed the second peak value that was 26.74; after that it dropped. The third peak value appeared until Sept 29, and that was 25.72; It dropped continuously since then, until Nov 14; And it rose slightly to 22.4 at Nov 29. After that it dropped to 16.72 rapidly (Figure 6A).

Compared to *GbDFR1*, the expression of *GbDFR2* gene showed 5 peak values in the growth process of *G. biloba*. The first expression peak appeared at the May 18 sampling point, its relative transcription level was 6.54; Then it dropped, and the first low valley appeared at June 13. After that it rose continuously, and then the second peak value appeared, it was 16.54, and this transcription level was also the peak of the year; Then it dropped continuously to the second low valley at Aug 13, that was 9.01; After that it rose to the third peak value at Aug 30, the relative transcription level was 10.8; The fourth peak value appeared at the Oct 17 sampling point, and it was 10.29; After that it dropped continuously to the fourth low valley at Nov. 14; And rose slightly to 6.39 at Nov. 29. Then it dropped to 2.05 rapidly (Figure 6B).

The expression level of *GbDFR3* gene showed 4 obvious peak values in the annual growth process of *Ginkgo* (Figure 6C). The first appeared at the July 18 sampling point, and the relative transcription level was 31.10; and then it dropped to the first low valley which appeared at Aug 1. Then it rose continuously to the second peak value that was 31.37; after a significant drop it rose again at Sept. 1. And the third peak value appeared until Sept 29, that was also the peak of the year, and it was 32.42; after that it dropped continuously. The fourth peak value appeared until Nov 29, and that was 24.84; after that it dropped to 17.53 rapidly at Dec. 10.

**Figure 7:**
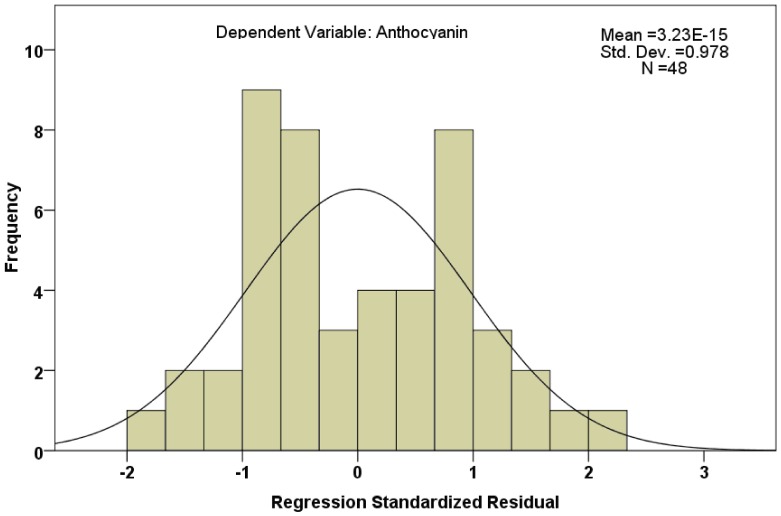
Histogram of regression standardized residual.

Table 2 and Table 3 showed the changes of the coefficient of determination of two fitted models, And it can be seen from the adjusted R^2^, that the proportion of explained variance of model 2 is much lager than that of model 1 of the total variance, with the variable *GbDFR2* opt-in. Table 4 showed the test results of coefficients of these two models, and T-test was adopted. It can be seen from the results, that two coefficients of independent variables of model 2 were statistically significant, the partial regression coefficient of *GbDFR3* was 0.124, and the standardized regression coefficient of *GbDFR3* was 0.981. The partial regression coefficient of *GbDFR2* was −0.1, and the standardized regression of *GbDFR2* was −0.595. We found through the comparison of the absolute values, that the contribution of *GbDFR3* to anthocyanin content was larger. The results of Table 5 reflected the test results of variables which were not selected in the models in the fitting progress of the multiple linear regression models. It can be seen from the results that the candidate variable *GbDFR2* which was not selected in the model was also in line with the inclusion criterion in model 1, so *GbDFR2* ought to be selected. The histogram of residuals was shown in Figure 7. It can be seen, that the residual distribution is relatively uniform, near normal distribution. This reflected that dependent variable follow a normal distribution. And in model 2, not-selected variable *GbDFR1* was greater than the inclusion criterion, so there was no necessary to analyze anymoreFinally the optimal equation we fitted was: Y = (1.739±0.334)+(0.124±0.017)X_3_+(−0.103±0.023)X_2_, among the formula, X_2_ and X_3_ indicated the relative transcription level of *GbDFR2* and *GbDFR3* respectively.

**Table 2:** The coefficient change of two model determination.

Model Summary^c^										
Model	R	R Square	Adjusted R Square	Std. Error of theEstimate	Change Statistics					Durbin-Watson
					R SquareChange	F Change	df1	df2	Sig. F Change	
1	0.587^a^	0.344	0.330	0.51320	0.344	24.151	1	46	.000	
2	0.737^b^	0.543	0.523	0.43302	0.199	19.612	1	45	.000	.673

a Predictors: (Constant), *Gb*DFR3.

b Predictors: (Constant), *GbDFR3*, *GbDFR2.*

c Dependent Variable: Anthocyanin.

**Table 3:** The inspection results of two models variance analysis.

ANOVA^c^						
Model		Sum of Squares	df	Mean Square	F	Sig.
1	Regression	6.361	1	6.361	24.151	0.000^a^
	Residual	12.115	46	0.263		
	Total	18.476	47			
2	Regression	10.038	2	5.019	26.767	0.000^b^
	Residual	8.438	45	0.188		
	Total	18.476	47			

a Predictors: (Constant), *GbDFR3.*

b Predictors: (Constant), *GbDFR3*, *GbDFR2.*

c Dependent Variable: Anthocyanin.

**Table 4:** Cofficient of the test results of the two models.

Coefficients^a^						
Model		Unstandardized Coefficients		Standardized Coefficients		
		B	Std. Error	Beta	t	Sig.
1	(Constant)	2.077	0.386		5.382	0.000
	*GbDFR3*	0.074	0.015	0.587	4.914	0.000
2	(Constant)	1.739	0.334		5.198	0.000
	*GbDFR3*	0.124	0.017	0.981	7.297	0.000
	*GbDFR2*	−0.103	0.023	−0.595	−4.429	0.000

a Dependent Variable: Anthocyanin.

**Table 5:** The multiple linear regression model of variable inspection.

Excluded Variables^c^						
Model		Beta In	t	Sig.	Partial Correlation	Collinearity Statistics
						Tolerance
1	*GbDFR1*	−0.038^a^	−0.170	0.866	−0.025	0.298
	*GbDFR2*	−0.595^a^	−4.429	0.000	−0.551	0.562
2	*GbDFR1*	0.254^b^	1.315	0.195	0.194	0.267

a Predictors in the Model: (Constant), *GbDFR3.*

b Predictors in the Model: (Constant), *GbDFR3*, *GbDFR2.*

c Dependent Variable: Anthocyanin.

## Discussion

DFR is a key enzyme in the anthocyanin biosynthetic pathway. Its catalytic reaction is an important point of regulation. The stereospecific reduction of C4 in dihydroflavonol catalyzed by DFR [32] is continued with reactions catalyzed by anthocyanin synthase (ANS) and flavonoid 3-O-glucosyltransferase (3GT) to synthesize a variety of anthocyanins [11].

### Bioinformatics Analysis of *GbDFRs*

In this study we isolated three homologous cDNA sequences of *DFRs* in *G. biloba*: *GbDFR1, GbDFR2* and *GbDFR3*. Bioinformatics analysis found that the proteins encoded by these genes have typical functional domains of DFR proteins, including a conserved NADPH binding motif “VTGAAGFIGSWLIMRLLERGY” [33], a substrate specificity selective domain (T135∼K160) and several specific loci of the conservative short-chain dehydrogenase/reductase family [34]. The number of amino acid residues in the DFR protein ranges from 330–345 in other species. The number of amino acids encoded by the *DFR* genes in *Ginkgo* is in accordance with this. It was also confirmed by analysis of proteins produced by prokaryotic expression that these three *DFR* genes in *Ginkgo* (*GbDFR1*, *GbDFR2* and *GbDFR3*) encode functional proteins, and that the number of amino acids in these functional proteins is consistent with predictions.

The substrate binding regions of DFRs from different species are highly conserved and the amino acid sequence of a DFR decides its substrate. Studies have found that amino acid residue 134 directly decides the substrate specificity. DFRs can be divided into three types according to differences at residue 134. First, there are the Asn-type DFRs, which have an asparagine residue (Asn) at this position. The DFR genes of *Vaccinium macrocarpon* and other plants belong to this type, as does *GbDFR3*. Second, residue 134 can be an aspartic acid residue (Asp). This type of DFR is called the Asp-type DFR, and it cannot convert DHK to leucopelargonidin effectively. The DFRs in *Petunia hybrida* and *Bromheadia finlaysoniana* belong to this type, as does *GbDFR2* in *Ginkgo*. The third type, in which residue 134 is neither Asn nor Asp, is called the non-Asn/Asp-type DFR [35], and *GbDFR1* belongs to this type. The most widely distributed DFR in plants is the Asn-type. DFRs in monocots all belong to Asn-type, but Asp-type DFRs are only distributed in some dicots. Only a few plants contain non-Asn/Asp-type DFRs. It can be supposed that the Asp- and non-Asn/Asp-types have evolved from the Asn-type. There are often different multiple DFRs in the same plant, such as in the legume *Lotus japonicus*, which has at least five distinct DFRs. *L. japonicus* DFR1 belongs to the non-Asn/Asp-type; it has a serine residue at position 134 and has no DFR activity. DFR2 and DFR3 in this species belong to the Asn-type, and show high activity with DHK and DHQ as a substrate, while DFR4 and DFR5 belong to the Asp-type, and their reaction rates for DHK are slower than for DHQ [36]. In *Medicago truncatula*, DFR1 belongs to the Asn-type, and DFR2 belongs to the Asp-type [11]. GbDFR1 can reduce DHQ, which is consistent with the fact that the location of the asparagine residue in DFR decides the substrate. Residue 134 of GbDFR2 is Asp, and thus DFR2 has very limited activity on DHK. Washburn *et al*. also found that residues 134 and 145 in the binding region directly affect the substrate specificity of the enzyme. The 134^th^ amino acid residue in most species is Asn or Asp, but in *V. macrocarpon* it is Val, and thus the substrate that this DFR enzyme can act on is limited to DHQ [11]. These examples illustrate that there are differences in the substrate selection of *GbDFRs*.

Along with DFR genes being isolated from different plants, research has been conducted on them at the nucleic acid and protein levels. Liu *et al*. constructed a phylogenetic tree including part of the species according to the homology of amino acid sequences of DFR [37]. According to the phylogenetic analysis, the homology among amino acid sequences of DFRs in monocotyledons is higher than it is in dicotyledons. Some researchers believe that the divergence of DFRs most likely occurred after the divergence between monocots and dicots, and that there may be homologous DFR genes in different plants [38]. Ostergaard *et al*. found proteins encoded by the *CRL1* and *CRL2* genes located on chromosome 2 of *Arabidopsis thaliana* that are highly homologous to the DFR superfamily. CRL1 and CRL2 belong to a separate branch of the DFR superfamily phylogenetically [39]. The phylogenetic tree indicated that Asn-type DFRs are widely distributed in plants, that and DFRs in monocotyledons are all Asn-type, while Asp-type DFRs are only distributed in some dicotyledons. The different DFRs in some species can also be divided into different groups. For example, in *M. truncatula*, DFR1 belongs to the Asn-type, and DFR2 belongs to the Asp-type [11]. Comparing DFRs in some important plants, we found that most DFRs of the plant kingdom are Asn-type. The number of Asp- and non-Asn/Asp-types is limited, and they are distributed distantly in the evolutionary tree. Thus, it can be supposed that the Asp- and non-Asn/Asp-types are evolved from the Asn-type.

There is also species specificity among nucleotide sequences in different species that possess DFR genes, the function of which may be in the transcription and translation stages. In Southern blot analysis of DFR1 in *Fagopyrum dibotrys*, the conservative probe hybridization results indicated that there is more than one homologous sequences of the *FdDFR1* gene in the genome of *F. dibotrys*. However, the specific probe hybridization results indicated that *FdDFR1* is a single copy gene [40]. The *Petunia hybrida* genome contains three DFR genes, located in chromosomes 2, 4 and 6 [41]. From this we can see that there are differences in DFR amino acid or nucleotide sequences in different species or in different types within the same species. The analysis of DFR homologous genes is therefore essential for the study of their functions at different growth stages or environment.

### Expression Analysis of *GbDFRs* in Different Organs

The spatial and temporal expression characteristics of DFR genes differ between species, developmental stages and plant organs. In *Saussurea medusa*, *SmDFR* is mainly expressed in floral organs, and its expression levels in leaf and root tissues is very low [13]. Two DFR genes in Asiatic hybrid lily are strongly expressed in colored tepals, anthers, filaments, pistils and red scales. The expression level of these two DFR genes rises during the growth and development of flowers, and reaches its maximum at the flowering stage. The DFR gene in spotless yellow tepals is only expressed in colored anthers and red scales. It can be seen that DFR genes are mainly expressed in anthocyanin-colored organs and their expression is coordinated with the generation of anthocyanins [42]. The expression of the DFR1 gene during the development of *A. thaliana* pollen tubes perhaps plays an important role in aspects of pollen maturation and the control of fertility [43]. Here, the high-level expression of *GbDFR2* was perhaps related to anthocyanin synthesis and the development of pollen in the *Ginkgo* male inflorescence.

There are five DFR genes in the *L. japonicus* genome that can produce six functional mRNAs, and have different tissue specificities. Shimada *et al.* detected the expression of DFR genes in *L. japonicus* fruits (seeds and pods), flowers, stems, leaves, roots and root nodules, and found that DFR1 was present in all of the these organs, DFR2 was mainly accumulated in all organs except leaves, DFR3 was only expressed in stems and leaves specifically, and DFR4 and DFR5 were expressed mainly in the aboveground parts except for some micro-expression in the roots [36]. *PtDFR1* and *PtDFR2*, two DFR genes cloned from poplar, are expressed differently in different tissues. These genes showed the highest expression level in root tissues and lower expression levels in young leaves. Specifically, *PtDFR1* showed the lowest expression in aging petioles, and *PtDFR2* showed the lowest expression in mature leaves [10]. In *Medicago truncatula*, the transcription level of *MtDFR1* during the development of leaves is significantly correlated with the level of anthocyanin accumulation. Transcripts of *MtDFR1* and *MtDFR2* are difficult to detect in non-photosynthetic parts or organs [11]. Compared to other plants, the flavonoid content is high in *G. biloba*. The strong expression of *GbDFR1* and *GbDFR2* in *Ginkgo* may be associated with the accumulation of flavonoids and anthocyanin synthesis in leaves. All three *GbDFRs* showed lower transcription levels in root tissues and stems. This may be similar to the expression of *MtDFR1* and *MtDFR2* in *M. truncatula*.

### Analysis of the Stress Expression of *GbDFRs*

When *Camellia sinensis* was subjected to wounding stress, the transcription level of its *CsDFR* gene first rose and then subsequently dropped, with the catechol content changing accordingly [7]. When *L. japonicus* was subjected to stress treatments (wounding stress), the expression of the *LjDFR* gene family showed big differences; *LjDFR1* and *LjDFR2* were inhibited at first, and after 12 h their transcription was restored to pre-treatment levels, but *LjDFR4* and *LjDFR5* were continuously inhibited by the wounding treatment. Ectopic expression analysis showed that *LjDFR2* was only expressed when the promoter was activated by transcription factors such as MYB, bHLH and WDR. This result indicated that each member of the *LjDFR* gene family is regulated independently [14]. Among the *DFR* genes in *Ginkgo*, wounding treatment only had a significant induction effect on *GbDFR2*, and weakly induced *GbDFR3*. This result implied that there must be activation elements for transcription factors such as MYB, bHLH and WDR in the *GbDFR2* gene promoter. This gene showed a similar response to the *LjDFR2* gene to outside pest and disease infection.

SA is a plant hormone and a signaling molecule for responses to outside environmental stresses and pest and disease infection [44]. The expression levels of flavonoid metabolism genes also rise dramatically when plants are infected by outside pests and diseases [45], [46]. In preliminary studies, it was found that SA treatment can significantly raise the expression levels of the *GbFLS* and *GbANS* genes [23]. In this study, we found that SA could significantly induce transcription of the *GbDFR2* gene to a high level, and sustain it for a long time. The *GbDFR2* gene also showed a high transcription level after external injuries. This indicates that *GbDFR2* is involved in the SA signal response pathway, which is induced by plant diseases, insect pests or wounding.

ABA can regulate the expression of related genes to help plants adapt to osmotic stresses such as frost, drought and high salinity [47]. In Litchi Chinensis Sonn, the expression of LcDFR in particular, was positively correlated with anthocynin concentrations in the pericarp [48]. ABA treatment can also improve the transcription levels of *G. biloba* flavonoid synthetic genes such as FLS [23] and CHI [8]. In the *GbDFR* gene expression analysis, we found that externally applied ABA could only induce the expression of *GbDFR1* and *GbDFR2*, and inhibited *GbDFR3* to a certain extent. Thus, *GbDFR1* is not only involved in the synthesis of anthocyanins in leaves, it may also be involved in the ABA signal-transduction pathway under osmotic stress, and appears to be a multi-functional gene. GbDFR2 is focused in the regulation of *Ginkgo* to adapt to a variety of adversities. *GbDFR3* was inhibited by externally applied ABA, and perhaps play a primary role in the synthesis of flavonoids and anthocyanins.

Ethylene (ETH) can prompote fruit ripening and coloring, and can regulate the expression of flavonoid metabolism-related genes [23], [26], [27]. It has been related with the accumulation of flavonoids in *Ginkgo* [24], [29]. Externally applied ETH can induce the expression of anthocyanin synthesis related genes, such as ANS/UFGT, in grape [49]. In *Ginkgo* cuttings seedlings treated with ETH, the transcription level of *GbANS* rose rapidly. In an in-depth study, it was found that there are ETH-cis-elements in the upper reaches of the *ANS* gene promoter. This is one of the reasons why *ANS* expression is easily induced by ETH [26]. The ETH-induced expression patterns of the of *GbDFR* genes was complicated: *GbDFR1* and *GbDFR3* responded significantly to ETH, but the increased expression level of *GbDFR3*, while being the highest, only lasted for a short time. The rise of the expression level of *GbDFR1* was slow but lasted for a longer time. *GbDFR2* was also induced by ETH, but it only rose significantly until 8 h after the treatment. These results suggest that *GbDFR2* perhaps participates in the *Ginkgo* defense against outside pests and diseases, *GbDFR3* play important roles in *Ginkgo* anthocyanin accumulation, and *GbDFR1* play a combination of both. the analysis of different tissues showed that the expression levels of *GbDFR1* and *GbDFR3* in leaves are very high. This result also corroborated the above speculation.

Low concentrations of ALA can raise rice, wheat and potato yields, especially when it is used in the wheat growth stage and rice young heading and flowering stages; the yield increase can reach 11–15% [50]. It is also noteworthy that ALA treatment can significantly promote the deepening of apple color and raise the anthocyanin content in apple peel [25]. ALA is a precursor in the biosynthesis of plant phytochrome [51]. Many studies have shown that low concentrations of ALA can promote photosynthesis in plants [25], [52], and phytochrome has a close relationship with flavonoid accumulation in *G. biloba* [53], [54]. Moreover, 100 mg/L ALA treatment can prompt a rise in the activities of *PAL*, *CHS*, and *CHI* in *Ginkgo* [55]. Thus, it can be supposed that ALA may have a promotive effect on *G. biloba* flavonoid accumulation, and that the transcription level increase is related to the rise of substrate level in this pathway.

*G. biloba* flavonoid metabolism is not only affected by intrinsic genetic factors. The effects of different light quality, light intensity and culture environment dimensions on flavonoid synthesis are also apparent [56]–[58]. Some studies have shown that the reason UV can induce the expression of flavonoid synthesis genes is because flavonoids can reduce UV damage to plants [59]. This response is related to UV-responsive elements in the upstream promoters of flavonoid metabolism related genes [8]. In fact, it has been reported that UV induces the expression of *G. biloba* flavonoid accumulation and related genes, such as *GbPAL* [27], *GbCHI* [8], *GbFLS* [23] and *GbANS* [26]. *GbDFR1* was strongly induced by UV-B, and its expression increase was rapid and lasted for a long time. *GbDFR2* was also induced by UV-B, but the increase in its transcription level was lower than that of *GbDFR1*. *GbDFR3* in the leaves of *G. biloba* was hardly induced by UV-B. Thus, it can be supposed that the genes that participate in the UV-stress response in *G. biloba* leaves are mainly *GbDFR1* and *GbDFR2*. Although *GbDFR3* is involved in anthocyanin synthesis, it may not be involved in UV-induced synthesis of these compounds.

### The Position of the *GbDFRs* in Flavonoids and Anthocyanin Synthesis

The *DFR* genes have great significance in flavonoid anabolism. Dynamic study of anthocyanins in *Prunus virginiana* leaves showed that DFR activity is related to anthocyanin content, and DFR may function in initiating the synthesis of anthocyanins [60]. A study by Kim *et al.* suggested that deletion mutants of the *DFR* gene in *Allium cepa* were the limiting factor that led to a lack of DFR activity and a drop in anthocyanin accumulation [61]. In a study where *Populus trichocarpa DFR* genes were transformed into tobacco, it was found that the anthocyanin content in *PtrDFR1*-positive tobacco plants was significantly higher than in the control. It was found in the *PtrDFR1* transgenic poplar that not only was anthocyanin synthesis increased, but tannic acid synthesis was also increased. However, *PtrDFR2* only improved the tannic acid accumulation in *PtrDFR2*-overexpressing poplar; there was no significant change in anthocyanin content [10]. Even in the same tissue, at different stages the transcription levels of *DFR* are quite different. In *C. sinensis* leaves at different development stages, the transcription level of *CsDFR* is significantly correlated with the catechol content. The transcription of *CsDFR* is greatest in the terminal bud, and gradually decreases from the first leaf to the fourth, where it is hardly expressed at all [7].

During the annual growth cycle of *G. biloba*, *GbDFR3* expression is significantly correlated with anthocyanin accumulation in leaves. We found that *GbDFR2* expression was negatively correlated with anthocyanin synthesis in *G. biloba*. Combined with tissue expression and induction analysis, We suppose that *GbDFR2* may be primarily responsible for tannic acid synthesis, and thus limits a certain amount of anthocyanin accumulation. Instead, *GbDFR1* probably correlated with resistance-related expression and anthocyanin accumulation in leaves.

## Conclusion

A fitted linear curve showed the best model for relating *GbDFR2* and *GbDFR3* with anthocyanin accumulation in leaves. Induced expression analysis showed GbDFR1 and GbDFR2 in Ginkgo may be specialized for environmental stress during plant evolution. Taken together, these results suggest that *GbDFR1* appears to be involved in environmental stress response, and *GbDFR3* likely has primary functions in the synthesis of anthocyanins, while GbDFR2 has the function of both. These data revealed unexpected properties and differences in three DFR proteins from a single species.

## Supporting Information

Figure S1 **Biosynthetic relationship of DFR to anthocyanidins, leuco-anthocyanidins, catechins, and condensed tannins (Xie et al., 2003).** CHI, chalcone isomerase; F3H, (2S)-flavanone 3-hydroxylase; F3′H, flavonoid 3-hydroxylase; F3′, 5′H, flavonoid 3′, 5′-hydroxylase; ANS, anthocyanidin synthase; GT, anthocyanidin glucosyl transferase; LAR, leucoanthocyanidin reductase.
(TIF)

Figure S2 **The full-length cDNA sequence and deduced amino acid sequence of** ***GbDFR1*** **gene.** The start condon (ATG), the stop (TAA) and putative polyadenylation signals are underline. Degenerate primers are indicated with “□”.
(TIF)

Figure S3 **The full-length cDNA sequence and deduced amino acid sequence of** ***GbDFR2*** **gene.** The start condon (ATG), the stop (TAA) and putative polyadenylation signals are underline. Degenerate primers are indicated with “□”.
(TIF)

Figure S4 **The full-length cDNA sequence and deduced amino acid sequence of** ***GbDFR3*** **gene.** The start condon (ATG), the stop (TGA) and putative polyadenylation signals are underline. Degenerate primers are indicated with “□”.
(TIF)

Figure S5 **Genomic blot analysis of** ***GbDFRs*** **.** Genomic DNA was digested with Sma I, Sac I, Eco V, Kpn I, Pvu II, Xma I, Dra I, Cla I and Hind III. The DNA blot was hybridized with the insert in the *GbDFRs* cDNA clone. Positions of molecular weight markers are shown on the left.
(TIF)

Figure S6 **SDS-PAGE gel and Western blot analysis of GbDFR1 expressed in** ***E.coli*** **BL21 (DE3).** After IPTG induction, *E.coli* BL21 cells containing pET28a-DFRs were grown at 30°C for 2 h. M, molecular marker; lane 1, protein of total cells without IPTG induction; lane 2, protein of total cells with IPTG induction for 30 min; lane 3, protein of total cells with IPTG induction for 60 min; lane 4, induction for 90 min; lane 5, induction for 2 h; lane 6, purified recombinant GbDFR1 protein with Nickel-CL agarose affinity chromatography and used for enzyme activity assay; Lane western blot, western blotting of the purified recombinant GbDFR1 protein with an anti-His-tag primary antibody probe.
(TIF)

Figure S7 **SDS-PAGE gel and Western blot analysis of GbDFR2 expressed in** ***E.coli*** **BL21 (DE3).** After IPTG induction, *E.coli* BL21 cells containing pET28a-DFR2 were grown at 30°C for 2 h. M, molecular marker; lane 1, protein of total cells without IPTG induction; lane 2, protein of total cells with IPTG induction for 30 min; lane 3, protein of total cells with IPTG induction for 60 min; lane 4, induction for 90 min; lane 5, induction for 2 h; lane 6, purified recombinant GbDFR2 protein with Nickel-CL agarose affinity chromatography and used for enzyme activity assay; Lane western blot, western blotting of the purified recombinant GbDFR2 protein with an anti-His-tag primary antibody probe.
(TIF)

Figure S8 **SDS-PAGE gel and Western blot analysis of GbDFR3 expressed in** ***E.coli*** **BL21 (DE3).** After IPTG induction, *E.coli* BL21 cells containing pET28a-DFR3 were grown at 30°C for 2 h. M, molecular marker; lane 1, protein of total cells without IPTG induction; lane 2, protein of total cells with IPTG induction for 30 min; lane 3, protein of total cells with IPTG induction for 60 min; lane 4, induction for 90 min; lane 5, induction for 2 h; lane 6, purified recombinant GbDFR3 protein with Nickel-CL agarose affinity chromatography and used for enzyme activity assay; Lane western blot, western blotting of the purified recombinant GbDFR3 protein with an anti-His-tag primary antibody probe.
(TIF)

Table S1 **Relative quantities of GbIRL1 mRNA at various time points post-treatment with Wounding(WOU), ultraviolet (UV-B), Abscisic Acid (ABA), Salicylic Acid (SA), Ethephon(ETH), 5-Aminolevulinic Acid (ALA).** Each sample was individually assayed in triplicate. ‘+’ shown represent the increasing transcription of fold change and ‘−’ shown represent the decreasing of *GbDFRs* transcription level.
(DOC)
