# Supplementary figures and images for: Molecular Cloning and Characterization of Three Genes Encoding Dihydroflavonol-4-Reductase from Ginkgo biloba in Anthocyanin Biosynthetic Pathway
Source: PLoS One. 2013 Aug 26;8(8):e72017. doi: 10.1371/journal.pone.0072017 (PMC3753345; doi:10.1371/journal.pone.0072017)

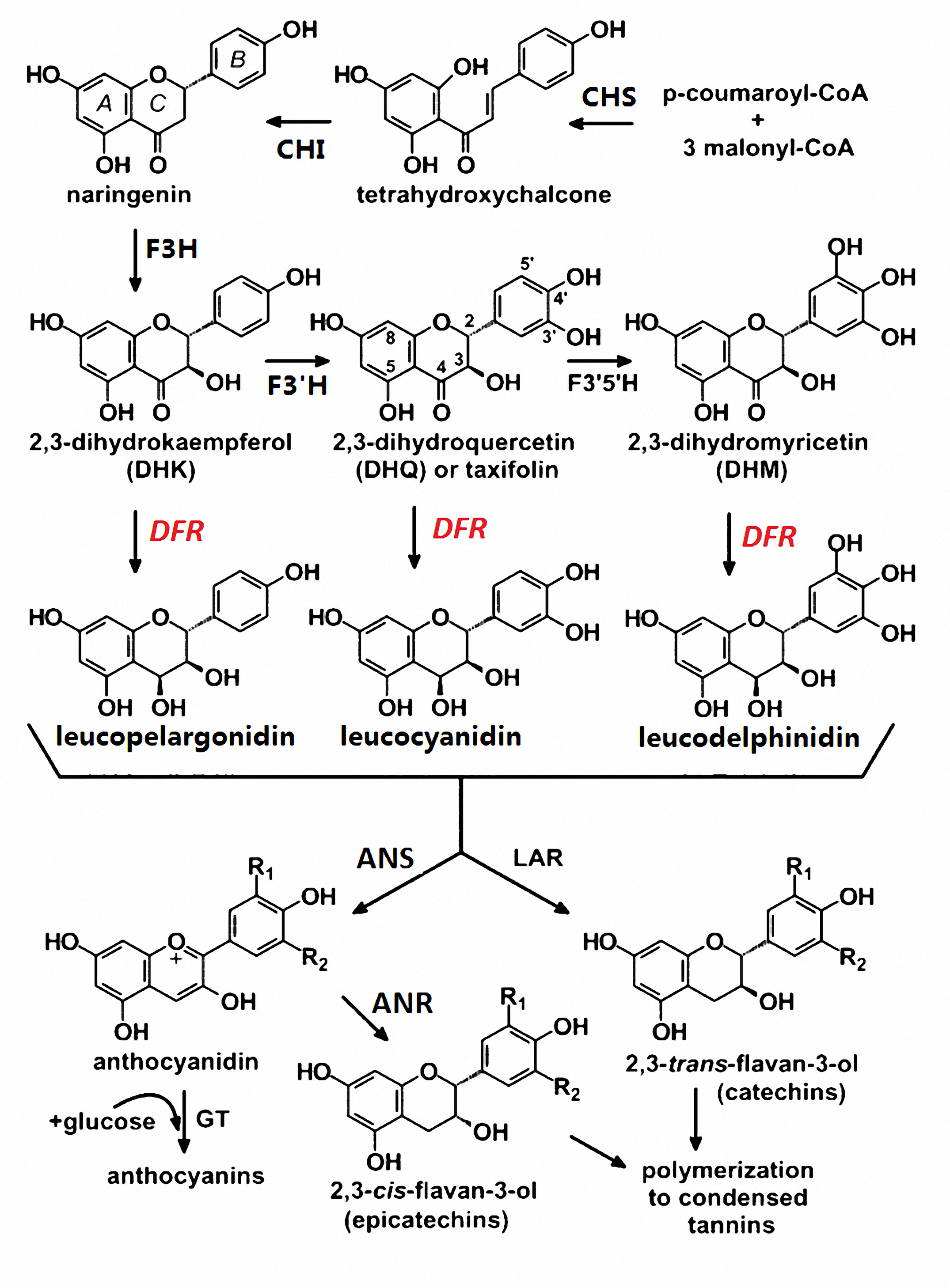

Supplement: Figure S1 — Biosynthetic relationship of DFR to anthocyanidins, leuco-anthocyanidins, catechins, and condensed tannins (Xie et al., 2003). CHI, chalcone isomerase; F3H, (2S)-flavanone 3-hydroxylase; F3′H, flavonoid 3-hydroxylase; F3′, 5′H, flavonoid 3′, 5′-hydroxylase; ANS, anthocyanidin synthase; GT, anthocyanidin glucosyl transferase; LAR, leucoanthocyanidin reductase. (TIF) [file pone.0072017.s001.tif]

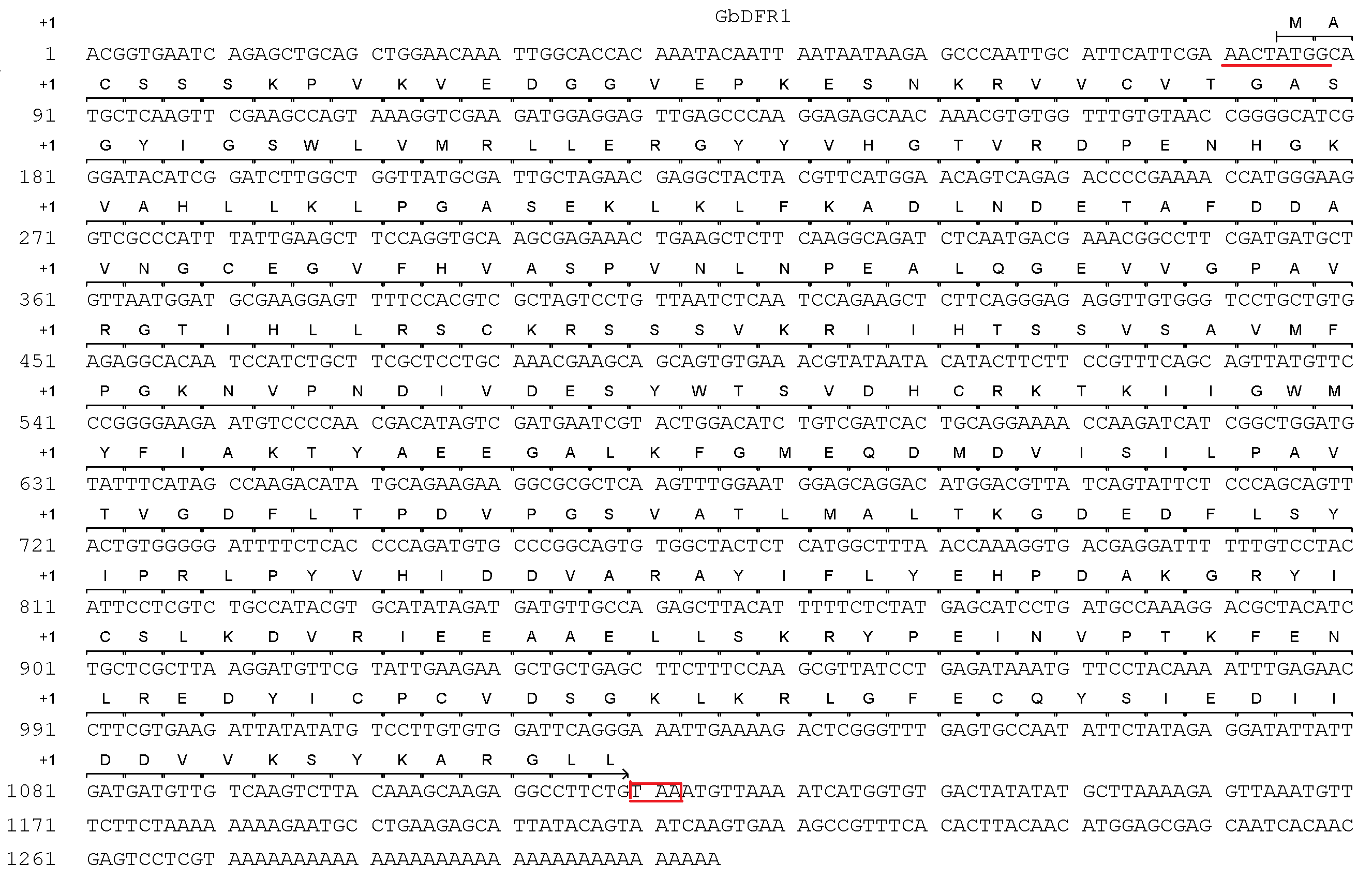

Supplement: Figure S2 — The full-length cDNA sequence and deduced amino acid sequence of GbDFR1 gene. The start condon (ATG), the stop (TAA) and putative polyadenylation signals are underline. Degenerate primers are indicated with “□”. (TIF) [file pone.0072017.s002.tif]

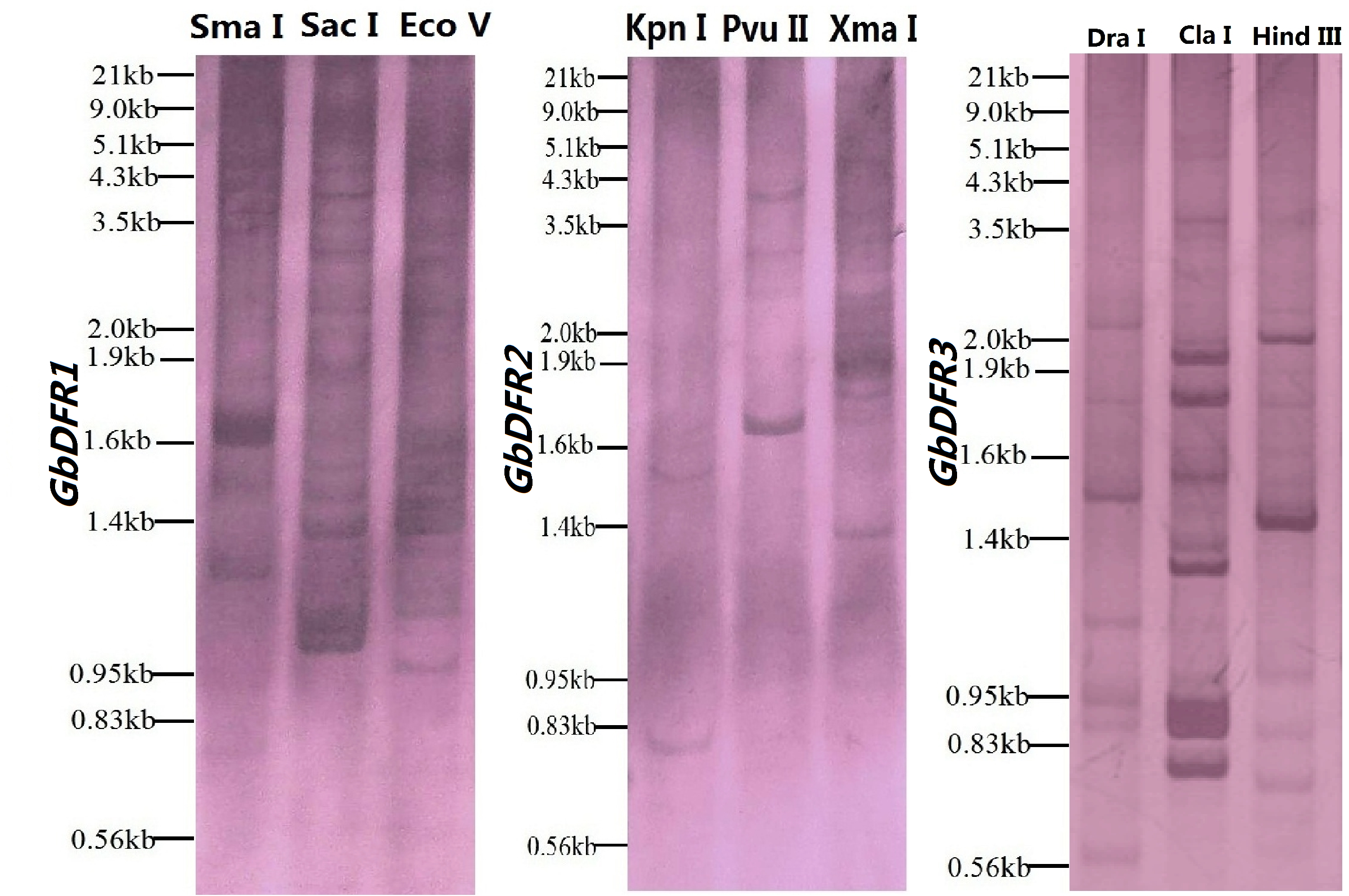

Supplement: Figure S5 — Genomic blot analysis of GbDFRs . Genomic DNA was digested with Sma I, Sac I, Eco V, Kpn I, Pvu II, Xma I, Dra I, Cla I and Hind III. The DNA blot was hybridized with the insert in the GbDFRs cDNA clone. Positions of molecular weight markers are shown on the left. (TIF) [file pone.0072017.s005.tif]

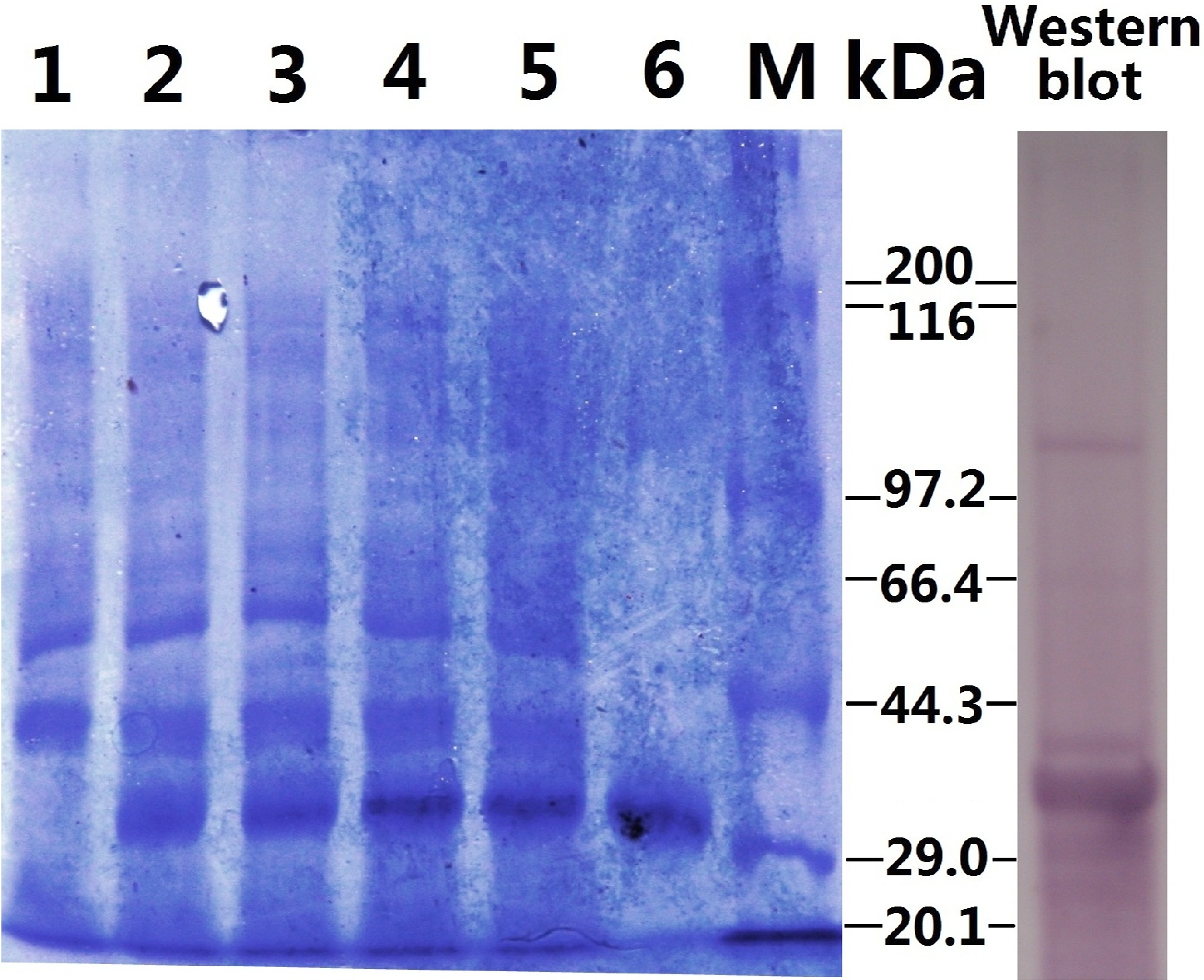

Supplement: Figure S6 — SDS-PAGE gel and Western blot analysis of GbDFR1 expressed in E.coli BL21 (DE3). After IPTG induction, E.coli BL21 cells containing pET28a-DFRs were grown at 30°C for 2 h. M, molecular marker; lane 1, protein of total cells without IPTG induction; lane 2, protein of total cells with IPTG induction for 30 min; lane 3, protein of total cells with IPTG induction for 60 min; lane 4, induction for 90 min; lane 5, induction for 2 h; lane 6, purified recombinant GbDFR1 protein with Nickel-CL agarose affinity chromatography and used for enzyme activity assay; Lane western blot, western blotting of the purified recombinant GbDFR1 protein with an anti-His-tag primary antibody probe. (TIF) [file pone.0072017.s006.tif]

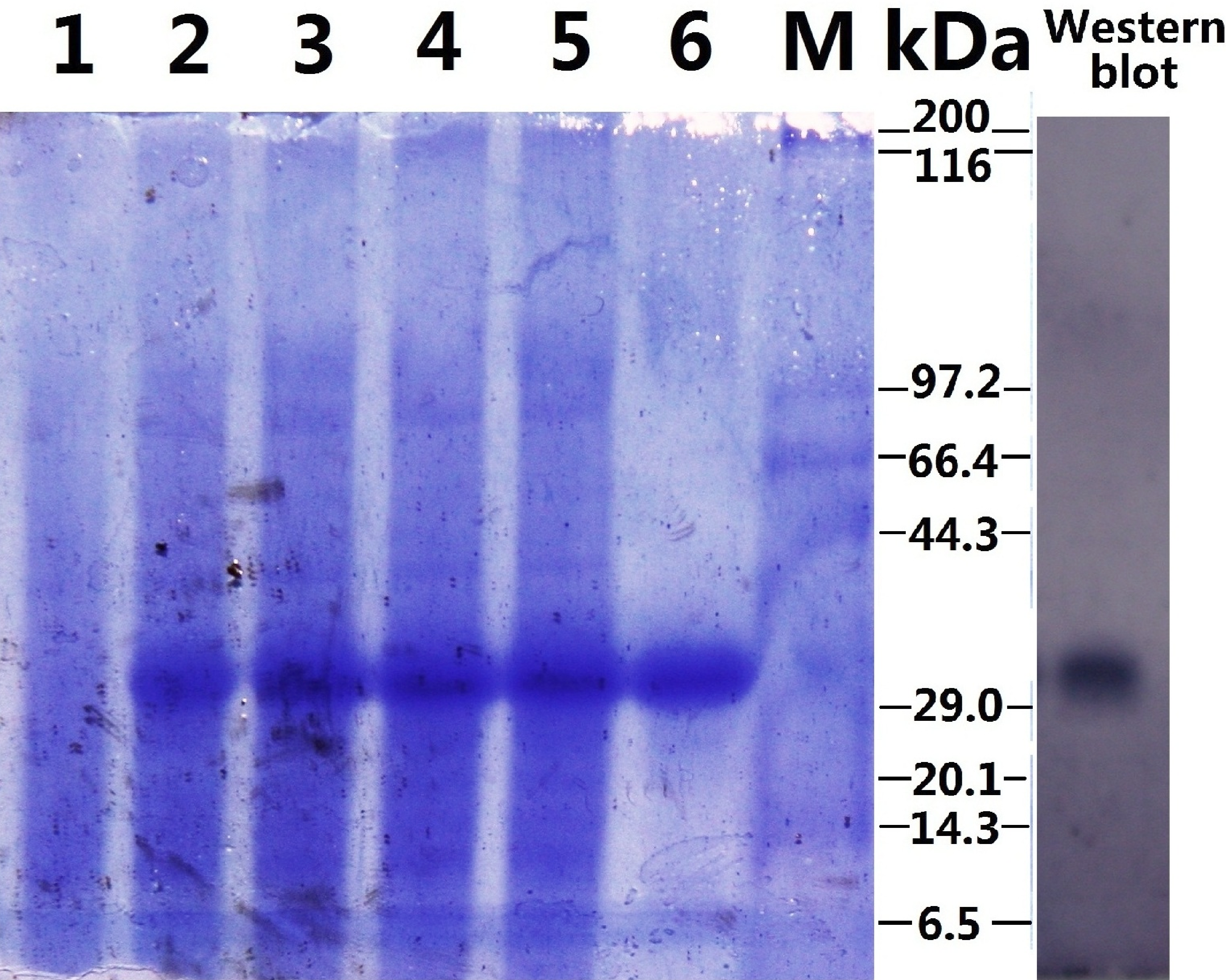

Supplement: Figure S7 — SDS-PAGE gel and Western blot analysis of GbDFR2 expressed in E.coli BL21 (DE3). After IPTG induction, E.coli BL21 cells containing pET28a-DFR2 were grown at 30°C for 2 h. M, molecular marker; lane 1, protein of total cells without IPTG induction; lane 2, protein of total cells with IPTG induction for 30 min; lane 3, protein of total cells with IPTG induction for 60 min; lane 4, induction for 90 min; lane 5, induction for 2 h; lane 6, purified recombinant GbDFR2 protein with Nickel-CL agarose affinity chromatography and used for enzyme activity assay; Lane western blot, western blotting of the purified recombinant GbDFR2 protein with an anti-His-tag primary antibody probe. (TIF) [file pone.0072017.s007.tif]

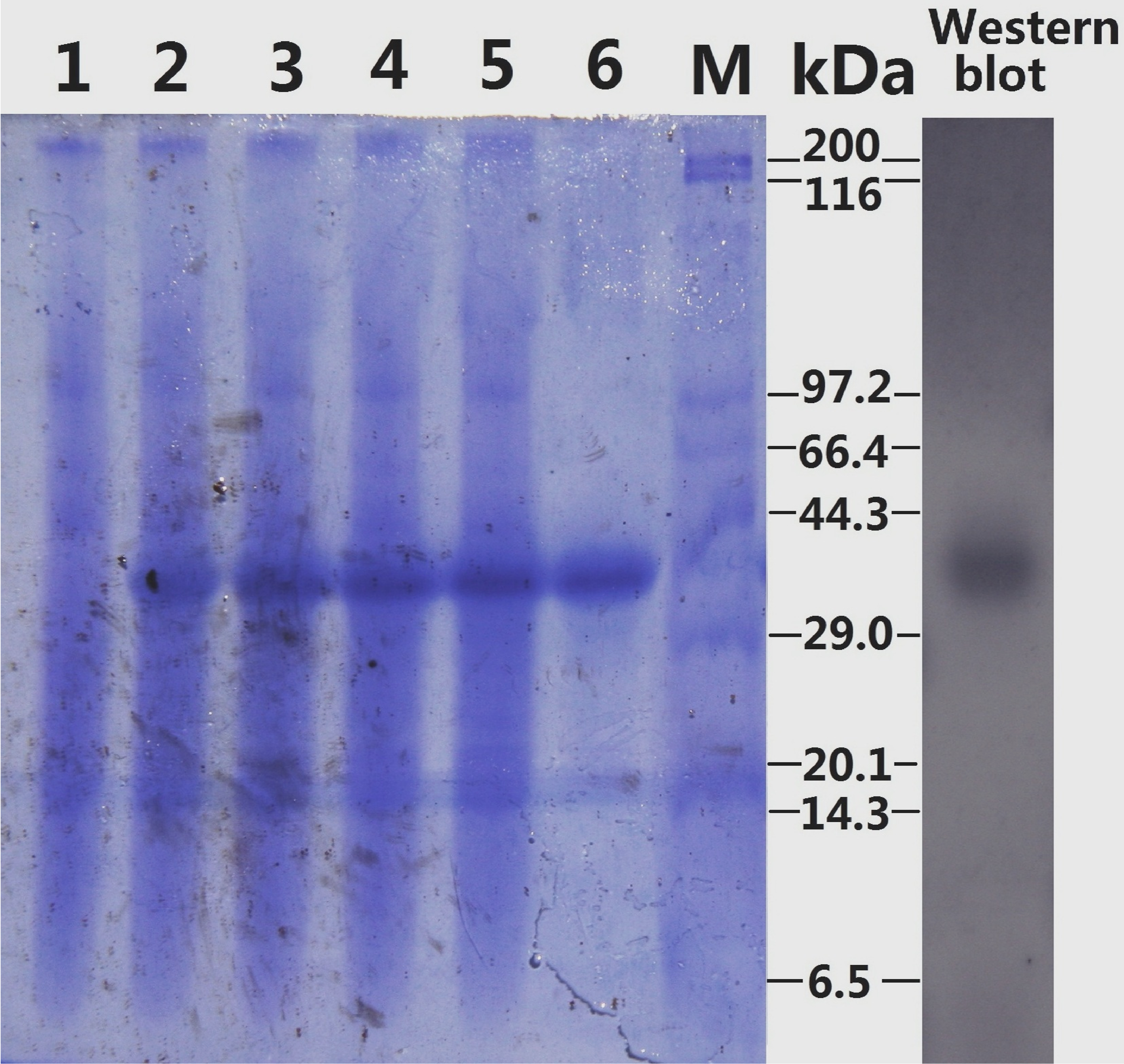

Supplement: Figure S8 — SDS-PAGE gel and Western blot analysis of GbDFR3 expressed in E.coli BL21 (DE3). After IPTG induction, E.coli BL21 cells containing pET28a-DFR3 were grown at 30°C for 2 h. M, molecular marker; lane 1, protein of total cells without IPTG induction; lane 2, protein of total cells with IPTG induction for 30 min; lane 3, protein of total cells with IPTG induction for 60 min; lane 4, induction for 90 min; lane 5, induction for 2 h; lane 6, purified recombinant GbDFR3 protein with Nickel-CL agarose affinity chromatography and used for enzyme activity assay; Lane western blot, western blotting of the purified recombinant GbDFR3 protein with an anti-His-tag primary antibody probe. (TIF) [file pone.0072017.s008.tif]
