# Supplementary material for: Molecular Cloning and Characterization of Three Genes Encoding Dihydroflavonol-4-Reductase from Ginkgo biloba in Anthocyanin Biosynthetic Pathway
Source: PLoS One. 2013 Aug 26;8(8):e72017. doi: 10.1371/journal.pone.0072017 (PMC3753345; doi:10.1371/journal.pone.0072017)
Supplement: Table S1 — Relative quantities of GbIRL1 mRNA at various time points post-treatment with Wounding(WOU), ultraviolet (UV-B), Abscisic Acid (ABA), Salicylic Acid (SA), Ethephon(ETH), 5-Aminolevulinic Acid (ALA). Each sample was individually assayed in triplicate. ‘+’ shown represent the increasing transcription of fold change and ‘−’ shown represent the decreasing of GbDFRs transcription level. (DOC) [file pone.0072017.s009.doc]

| Different DFR genes | Treatment | Time of treatment (hours) | | | | | |
| --- | --- | --- | --- | --- | --- | --- | --- |
|  |  | 4 | 8 | 12 | 24 | 48 | 96 |
| GbDFR1 | CK | + | + | + | + | + | + |
|  | ALA | + | ++ | ++ | +++ | +++ | ++ |
|  | UV-B | + | +++++ | ++++++ | +++++ | ++++ | +++ |
|  | ABA | +++ | +++ | +++ | +++ | ++++ | ++ |
|  | WOU | + | + | + | + | + | + |
|  | ETH | ++ | ++ | +++ | +++ | ++++ | ++++ |
|  | SA | + | - | -- | - | - | + |
| GbDFR2 | CK | + | + | + | + | + | + |
|  | ALA | + | + | + | + | + | + |
|  | UV-B | + | ++ | +++ | ++ | + | + |
|  | ABA | +++ | ++++ | +++++ | +++ | +++ | + |
|  | WOU | + | +++ | +++++ | ++++++ | ++ | + |
|  | ETH | + | +++ | ++ | + | + | + |
|  | SA | +++ | ++++ | ++++++ | ++++++ | +++++ | ++ |
| GbDFR3 | CK | + | + | + | + | + | + |
|  | ALA | + | + | + | + | + | + |
|  | UV-B | + | + | + | + | + | + |
|  | ABA | + | - | -- | + | - | + |
|  | WOU | + | ++ | +++ | + | + | + |
|  | ETH | +++ | ++++ | +++++ | ++++++ | ++ | ++ |
|  | SA | + | + | + | + | + | + |
